# Study on the effect of sevoflurane on the cognitive function of aged rats based on the activation of cortical microglia

**DOI:** 10.1002/ibra.12010

**Published:** 2021-12-09

**Authors:** Jun‐Jie Zhou, Chao Zhang, Xin‐Xin Yang, Xiao‐Xi Zhang, Nai‐Xin Zhang, Xu Fang, Yu‐Hang Zhu, De‐Xing Liu, Shan Xu, Mei‐Qi Xu, Zhao‐Qiong Zhu

**Affiliations:** ^1^ Department of Anesthesiology Affiliated Hospital of Zunyi Medical University Zunyi Guizhou China; ^2^ College of Animal Science/Institute of Agro‐Bioengineering and Key Laboratory of Plant Resource Conservative and Germplam Innovation in Mountainous Region (Ministry of Education) Guizhou University Guiyang Guizhou China; ^3^ School of Foreign Languages Zunyi Medical University Zunyi Guizhou China; ^4^ Soochow University Medical College Suzhou Jiangsu China

**Keywords:** argininase‐1, cognitive dysfunction, inducible nitric oxide synthase, inflammatory factor, ion calcium connectors molecule, microglia

## Abstract

Postoperative cognitive dysfunction (POCD) is a common clinical manifestation that is a severe complication characterized by decreased learning ability and deterioration of memory following anesthesia and surgery. However, the precise mechanisms of POCD are not completely understood. Rats were divided into blank control group (Con, *n* = 12) and sevoflurane group (Sev, *n* = 12). Morris water maze test was performed to evaluate the ability of learning and memory in two groups of rats; immunohistochemical staining was used to detect the expression of ion calcium‐binding adaptor molecule‐1 (Iba‐1) in rat prefrontal cortex (PFC); Western blot analysis was applied respectively to investigate Iba‐1, inducible nitric oxide synthase (iNOS), arginase‐1 (ARG1), inflammatory cytokines interleukin‐1β (IL‐1β), and tumor necrosis factor‐α (TNF‐α) expression; The expression of iNOS, ARG1, IL‐1β, and TNF‐α in sera of rats was detected by enzyme‐linked immunosorbent assay. We found that sevoflurane induced learning and memory impairment assessed by morris water maze test, anesthesia up‐regulated the expression of iNOS, IL‐1β and TNF‐α inflammasome in microglia, as indicated by increased activation of Iba‐1 and reduced the level of ARG1 in the PFC. We conclude that the cognitive function of rats after inhaling anesthesia was likely associated with M1/M2 polarization of microglia, which was triggered by sevoflurane.

## Graphical abstract

After sevoflurane intervention, microglia in the prefrontal cortex of aging model rats were activated. M1 microglia secrete and release interleukin‐1β and tumor necrosis factor‐α to promote the occurrence of inflammatory response. However, the release of arginase‐1 by protective M2 microglia was significantly inhibited.

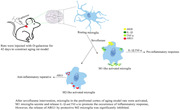

## 1 INTRODUCTION

Postoperative cognitive dysfunction (POCD) has become an important public health issue. It is also a common complication after surgery and anesthesia in the elderly. POCD is characterized by progressive hypomnesia, altered consciousness, personality change, or executive functions deterioration. Animal studies have shown that sevoflurane‐induced toxicity elicits apoptosis of rat hippocampal neurons and causes a cognitive deficit in aged rats.^1^ It needs to be noted that there was little neuroinflammation or cognitive impairment in the younger animals treated with the same conditions. Aging is an independent risk factor of cognitive dysfunction. Most neurodegenerative disease occurs primarily in the elderly population.^2^ Much data from animal and human studies show that neuroinflammation from either surgery or anesthesia plays a key role in the pathophysiological mechanism of POCD. Proinflammatory cytokines interleukin‐1β (IL‐1β) and tumor necrosis factor‐α (TNF‐α) have been shown involved in brain inflammation. Yet the specific relationship between inflammation and POCD remains unknown.^3^, ^4^

Microglial cells are the immune cells in the brain, which serve the role of immunity and host defense. They also provide immune surveillance in the central nervous system (CNS).^5^ Studies have shown that microglial cells are involved in the pathology of neurodegenerative disease. Microglia are activated rapidly after external stimulation or injury and play a dual role in the stress response. Activation of microglial cells induces the expression of proinflammatory factors and produces inflammatory responses. Emerging evidence have shown that activated microglia could be divided into “classic” or “alternative” activated populations, also known as M1 inflammatory microglia and M2 anti‐inflammatory microglia.^6^ However, the molecular mechanism of sevoflurane‐induced cognitive impairment in rats by activating microglia needs to be explored in the future. Moreover, whether sevoflurane impacts microglia activation and polarization remain unknown. Based on the above theory, in this study, we used d‐galactose to induce the aging rat model to observe the activation and expression of microglial cells in the prefrontal cortex (PFC) of aged rats after sevoflurane inhalation and to explore the possible pathological mechanism of POCD induced by sevoflurane, and to provide a reference for preventing and treating POCD.

## 2 MATERIALS AND METHODS

### 2.1 Animals

Twenty‐four male Sprague‐Dawley (SD) rats (Grade SPF, 350–400 g) were purchased from Changsha Tianqin Biotechnology Co. Ltd. The animals were housed under controlled conditions of 25 ± 2°C, relative humidity of 40 ± 20%, and 7:00–19:00 light, 19:00–7:00 darkness, as well as with free access to food and water. This study has been approved by the Ethics Committee of Zunyi Medical University.

### 2.2 Experimental materials

d‐Galactose (Solarbio, D8310); sevoflurane (Lunambert Pharmaceuticals Co. Ltd., 65190902); Pentobarbital (Merck, P11011); BCA Kit (SolarBio, PC0020); anti‐Iba‐1 antibody (Abacm, ab178846); anti‐iNOS antibody (Novus, NB300‐605); anti‐ARG1 antibody (Novus, NB100‐59740); anti‐IL‐1β antibody (Bioss, BS‐20449R); anti‐TNF‐α antibody (Abacm, ab205587); horseradish peroxidase labeled goat anti‐rabbit IgG (Abacm, ab205718); β‐actin antibody (Proteintech, 20536‐1‐AP); Maker (Thermo, 26616); Western HRP luminescent substrate (Merck, WBKLS0100); DAB Chromogenic Kit (ZLI‐9018); Rat ELISA Kit (Shanghai Jianglai Biology); MT‐200 Morris Water Maze (Chengdu Taimeng Technology Co. Ltd.); anesthesia machine (Drager Julian); anesthesia gas monitor (Drager Medical GmbH); enzyme standard instrument (Thermo); electrophoresis apparatus (Biorad); microscope (OLYMPUS, BX63).

### 2.3 Aging rat model

**Figure 1:**
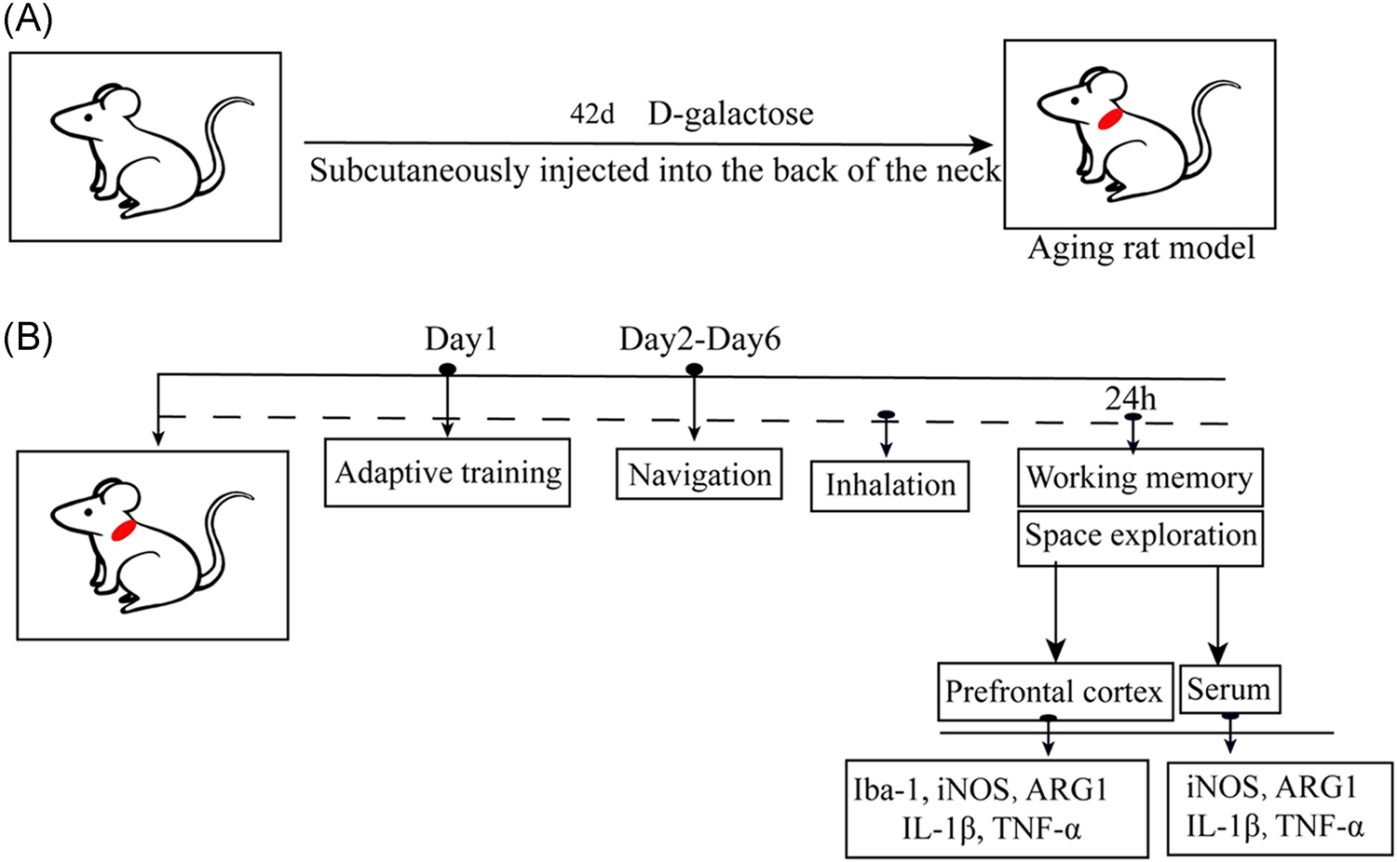
Flow chart of the experiment. (A) After 42 days of d‐galactose injection, the aging rat model was successfully established. (B) All rats were tested by Morris water maze, the first day is adaptive training, followed by positioning navigation test from the second day to the sixth day. Twenty‐four hours after the inhalation intervention for all rats, the prefrontal cortex of rats was collected, and Iba‐1, iNOS, ARG1, IL‐1β, and TNF‐α were detected. iNOS, ARG1, IL‐1β, and TNF‐α were detected in rat serum. ARG1, arginase‐1; IL‐1β, interleukin‐1β; iNOS, inducible nitric oxide synthase; TNF‐α, tumor necrosis factor‐α [Color figure can be viewed at wileyonlinelibrary.com]

In this study, the aging rat model was established according to the metabolic disorder theory of aging.^7^ The aging rat model was created by subcutaneous injection of 125 mg/kg/day^−1^ d‐galactose in the neck and back of rats for 42 days (Figure 1A).

### 2.4 POCD model

**Figure 2:**
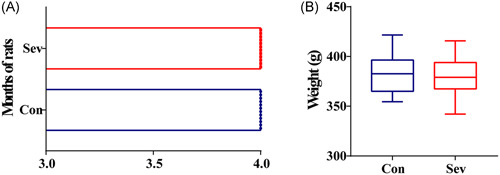
Age and weight of rats. (A) There was no significant difference in the age of the two groups of rats (*n* = 12). (B) There was no significant difference in the weight of rats in the two groups (*n* = 12) [Color figure can be viewed at wileyonlinelibrary.com]

The control group was given simple gas inhalation (1 L/min air + 3 L/min O_2_) for 6 h. The sevoflurane group was given gas inhalation and a 3.2% concentration of sevoflurane for 6 h (Figure 1B). It should be emphasized here that we were able to compare the age and weight of all the experimental animals and there was no significant difference between the two groups of rats (Figure 2A,B), suggesting that all the data in the subsequent experiments were highly reliable.

### 2.5 Morris water maze test (MWM)

Phase 1 of the experiment: 24 h after the injection of d‐galactose, the rats in the two groups were placed in MWM, respectively, for adaptive training and free swimming for 120 s. On the 2–6 days, a round black pool (120 cm in length and 60 cm in depth) was filled with water (25 ± 2°C) to a depth of 30 cm. A certain amount of milk powder was mixed in the water to make the water opaque. A circular platform (10 cm in diameter) was fixed in the third quadrant and submerged 1 cm below the water level of the platform as long as the platform was not visible. All rats were bathed once in each quadrant of a pool for 5 consecutive days. During each training, rats were randomly placed in the pool facing the basin wall and placed underwater. Rats were allowed to swim and discover the hidden platform within 120 s. Rats that could not find the platform were guided to stop for 20 s, and then the experiment was carried out in the next quadrant. The average calculated values of the four quadrants were calculated as the statistical results of the escape latency of rats on the same day. Phase 2 of the experiment: 24 h after awakening from anesthesia, space exploration tests were conducted on rats in each group. Rats were evacuated from the platform, entered the water from the first quadrant and swam for 120 s. The number of times each rat swam across the original platform was recorded. Phase 3 of the experiment: the platform was moved from the original position to the first quadrant. The rats in the third quadrant to the bowel wall were placed into the water. The rats were guided to find the platform in 120 s and stayed there for 30 s. The rats were then taken out of the water and were placed back into the water again after 1 min. Their escape latency time was recorded.

### 2.6 Immunohistochemical (IHC) staining

At the end of the behavior study, six rats were taken from each group. Immediately after pentobarbital anesthesia, brain tissues were removed and placed in 4% paraformaldehyde solution on ice. The brain tissues were fixed for 36 h at room temperature, embedded in paraffin and sliced. Before ethanol hydration, the slices were soaked in C_8_H_10_ twice, each time for 10 min. Before being soaked in H_2_O_2_ for 10 min, the slices were washed with PBS three times. Next, they were cleaned with distilled water for 10 min, PBS for 10 min, EDTA for antigen repair, sealed in 10% normal goat serum for 2 h and added with ionic calcium joint molecules (Iba‐1, 1:500) of the primary antibody after being cleaning. Then the slices were incubated in a wet box in the refrigerator for 12 h. The next day, they were cleaned and dripped with antibody working solution by PBS after rewarming. They were incubated for 2 h at 37°C and cleaned with PBS three times. DAB working solution that was prepared 10 min in advance was added. The reaction degree was controlled under the microscope. After washing with PBS three times, the slices were stained with hematoxylin. Gradient ethanol was dehydrated. Xylene was transparent. After drying, the plates were sealed. Five fields of view were randomly chosen to take pictures under the optical microscope.

### 2.7 Western blot method

After the behavior study, the PFC of the remaining rats was taken on ice after anesthesia. The residual blood was washed with precooled saline. RIPA + PMSF (100:1) was added to homogenate to mix the tissue. After high‐speed centrifugation for 15 min, the supernatant was absorbed. The BCA method was used to determine the protein concentration. The transfer membrane was separated by gel electrophoresis and was sealed in defatted milk for 2 h. The primary antibody working medium was added, and after washing with TBST the next day, it was added and incubated in the secondary antibody chamber for 1 hour, exposure with Western HRP luminescence substrate. Protein expression was detected by Image J.

### 2.8 Enzyme‐linked immunosorbent assay (ELISA)

After anesthesia, the rats were fixed on the operating table. Abdominal aortic blood was taken and injected into the procoagulant tube. After placement for 10 min at room temperature, the upper serum was removed and transferred to the EP tube for later use. Subsequent experiments were carried out in strict accordance with the additional instructions of the kit.

### 2.9 Statistics analysis

SPSS18.0 statistical software was used to process the measurement data. The water maze was compared by repeated measurement and single factor spherical test. The remaining data were tested by an independent sample *t*‐test. The correlation analysis was performed using the Pearson correlation coefficient. The measurement data were expressed as mean ± standard deviation. *p* < 0.05 was considered to be statistically significant.

## 3 RESULTS

### 3.1 Reduced learning and memory in rats were associated with sevoflurane inhalation

**Figure 3:**
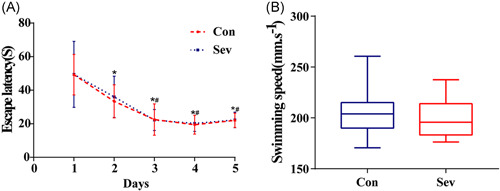
Positioning navigation test results. (A) The learning and memory abilities of the two groups were at the same baseline, *n* = 12. (B) There was no significant difference in swimming speed between the two groups, *n* = 12. *p < 0.05 versus Day 1, #*p* < 0.05 versus Day 2 [Color figure can be viewed at wileyonlinelibrary.com]

**Figure 4:**
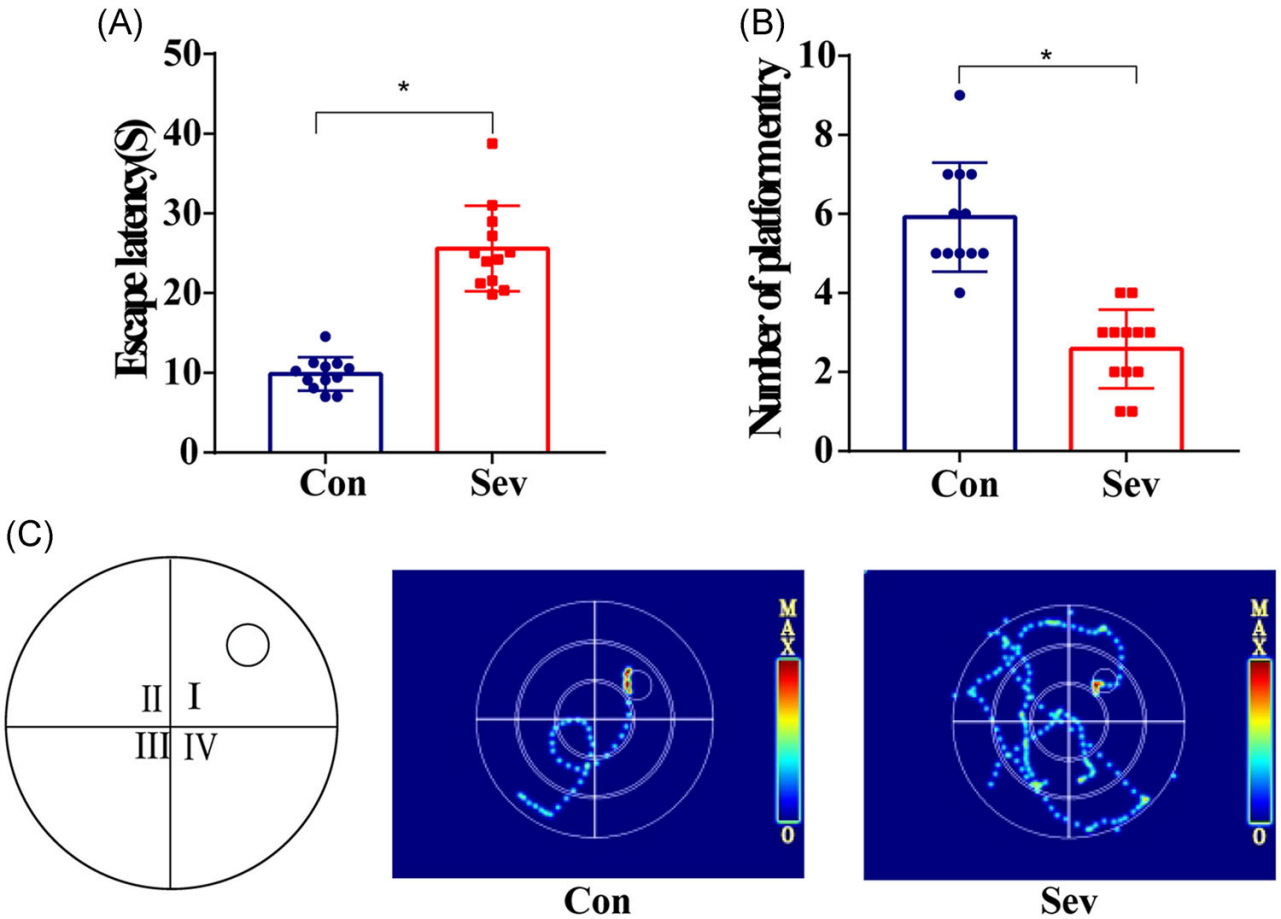
Working memory test results. (A) Escaping latency statistics of rats in the Con group and the Sev group (*n* = 12). (B) Statistical results of The Times of rats in the Con group and the Sev group crossing the original platform (*n* = 12). (C) Illustrations of sailing routes recorded by two groups of rats in a working memory test. *p < 0.05 versus Con group [Color figure can be viewed at wileyonlinelibrary.com]

The learning skills of each rat were expressed by the escape latency after training for days. As experimental days went on, the time of escaping latency in the last 3 days of the two groups was gradually shortened and stable. There is no statistical difference between the two groups (Figure 3A, *p* > 0.05). Similarly, by analyzing the swimming speed of each group of rats, there is no significant difference in swimming speed between the two groups (Figure 3B, *p* > 0.05), which indicates that the baseline of learning skills of the two groups of animals is consistent. In the space exploration experiment, we observed that the times of swimming across the platform in the Sev group were significantly less than those in the Con group and in the test of working memory. The escape latency of Sev group rats was significantly longer than that of the Con group (Figure 4A,B, *p* < 0.05). Moreover, the movement trajectory of rats in the Sev group was slightly more chaotic than that in the Con group (Figure 3C, *p* < 0.05), which indicated that sevoflurane can obviously reduce the learning and working memory of aging model rats.

### 3.2 Sevoflurane increases the number and activation degree of microglia in PFC of rats

**Figure 5:**
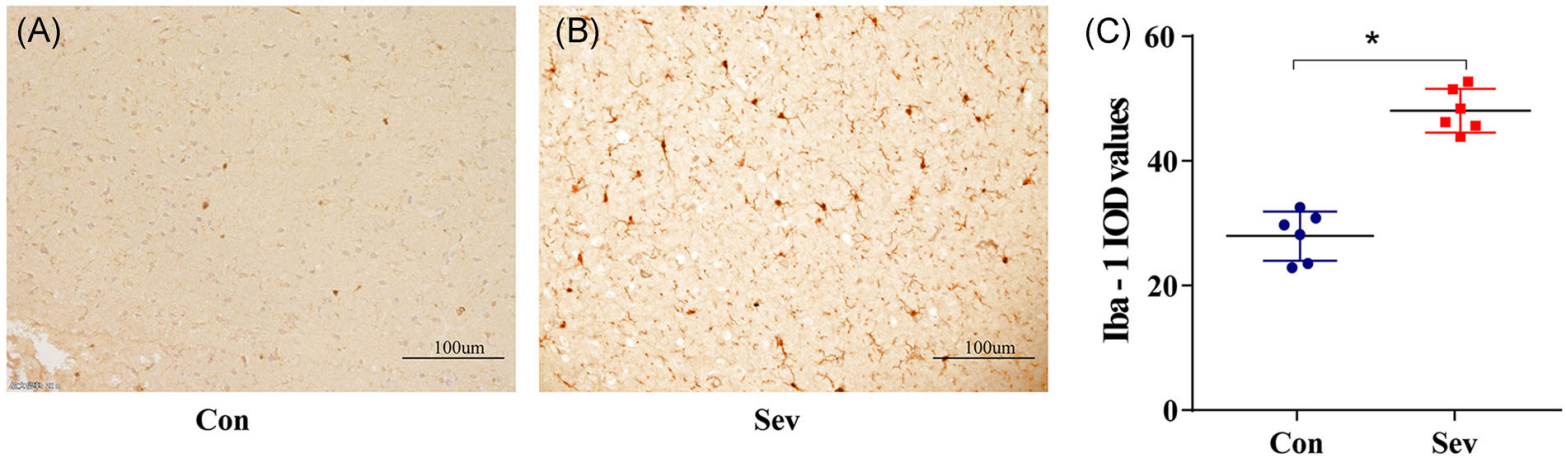
Immunohistochemical results. (A and B) Microscopically, this is representative immunohistochemical (IHC) staining image with dark brown Iba‐1‐positive cells, image size ×200 magnification, specifications for 100 μm, *n* = 6. (C) IHC statistical results of the two groups of rats, the results obtained from the average values of each integrated optical density. *p < 0.05 versus Con group [Color figure can be viewed at wileyonlinelibrary.com]

The results of IHC showed that microglia with small process branches were scattered in the PFC of rats in the Con group. Compared with the Con group, the number of Iba‐1‐positive cells in the Sev group increased significantly. The morphology of microglia changed like an amoeba, and the integrated optical density of Iba‐1 increased significantly (Figure 5C, *p* < 0.05). It is suggested that sevoflurane could activate microglia in the PFC of rats.

### 3.3 Sevoflurane upregulates the expression level of Iba‐1, iNOS, ARG1, inflammatory factor IL‐1β, and TNF‐α protein in rats’ cortex

**Figure 6:**
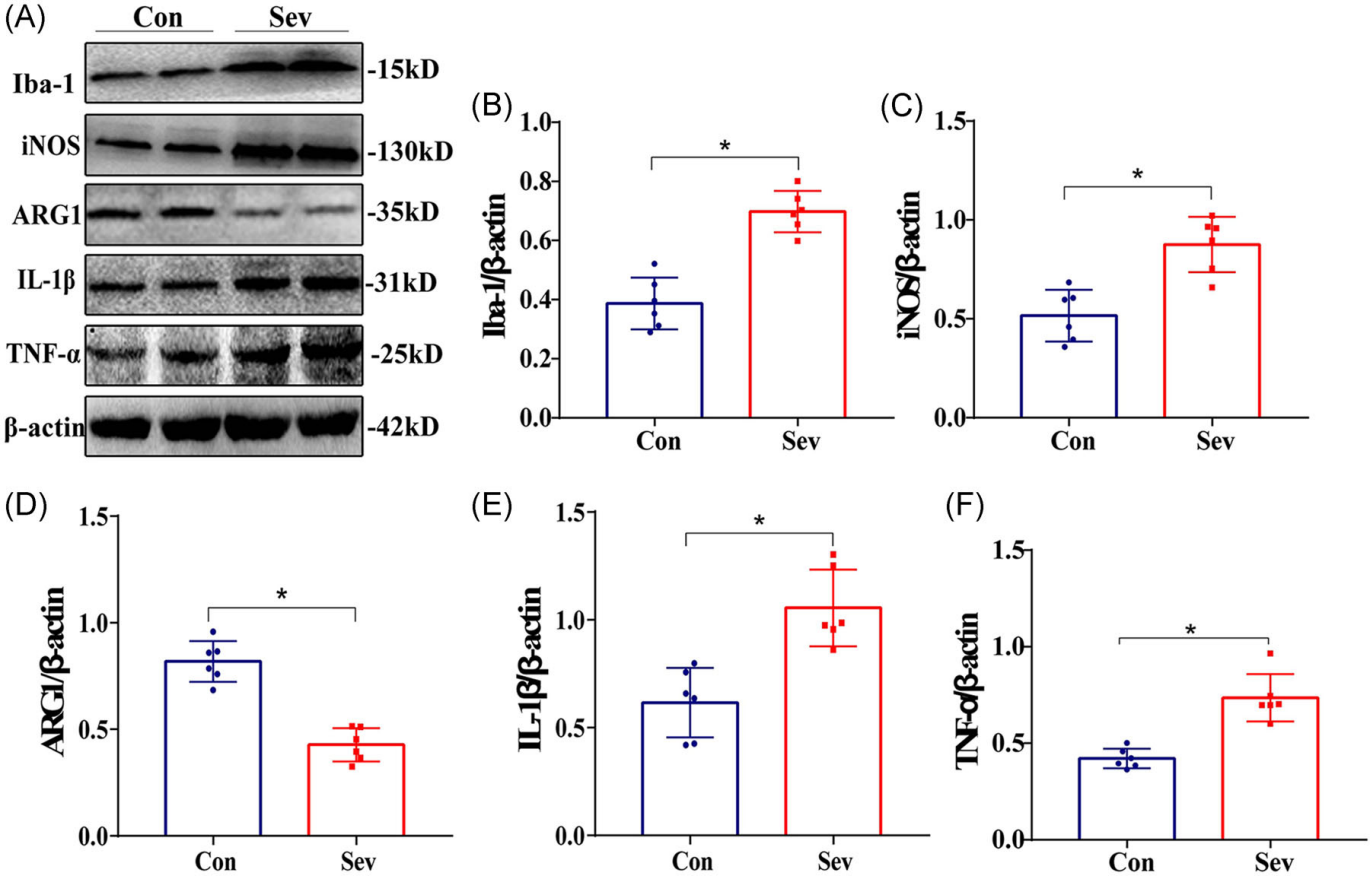
Western blot results. (A) Representative Western blot and quantitative analysis of protein levels of Iba‐1, iNOS, ARG1, IL‐1β, and TNF‐α in PFC tissues, *n* = 6. (B) Relative level of Iba‐1 protein in PFC (fold change relative to β‐actin protein level). (C) The relative level of iNOS protein in PFC (fold change relative to β‐actin protein level). (D) The relative level of ARG1 protein in PFC (fold change relative to β‐actin protein level). (E) The relative level of IL‐1β protein in PFC (fold change relative to β‐actin protein level). (F) The relative level of TNF‐α protein in PFC (fold change relative to β‐actin protein level). *p < 0.05 versus Con group. ARG1, arginase‐1; IL‐1β, interleukin‐1β; iNOS, inducible nitric oxide synthase; PFC, prefrontal cortex; TNF‐α, tumor necrosis factor‐α [Color figure can be viewed at wileyonlinelibrary.com]

Compared with the Con group, the expression of Iba‐1 protein in the cortex of the Sev group increased, which was consistent with IHC results. In addition, the expressions of iNOS, IL‐1β, and TNF‐α protein were significantly upregulated, while the expression of ARG1 protein was significantly downregulated (Figure 6, *p* < 0.05). The results suggest that sevoflurane can cause CNS inflammation, which may be related to the decrease of learning and working memory ability caused by sevoflurane in rats.

### 3.4 Sevoflurane can increase the level of peripheral inflammatory factors

The results of ELISA testing iNOS, ARG1, and inflammatory factors IL‐1β and TNF‐α showed that the content of each index was calculated in strict accordance with the steps of the kit. Compared with the Con group, the content of iNOS, IL‐1β, and TNF‐α in the Sev group increased significantly, while the content of ARG1 decreased significantly (Table 1, *p* < 0.05). It suggested that sevoflurane could increase the level of peripheral inflammatory factors.

**Table 1:** The PFC levels of inflammatory factors in the rats of each group (ng/ml, mean ± SD, *n* = 12)

Group	iNOS	ARG1	IL‐1β	TNF‐α
Con	68.24 ± 15.34	77.19 ± 10.61	42.37 ± 9.48	50.39 ± 7.67
Sev	86.47 ± 10.29*	54.31 ± 9.52*	71.49 ± 11.09*	94.13 ± 11.79*

Abbreviations: ARG1, arginase‐1; IL‐1β, interleukin‐1β; iNOS, inducible nitric oxide synthase; PFC, prefrontal cortex; TNF‐α, tumor necrosis factor‐α.

*At 0.0001 significant horizontal (bilateral) correlation, and this is automatically generated by SPSS statistical software.

### 3.5 Correlation test

**Figure 7:**
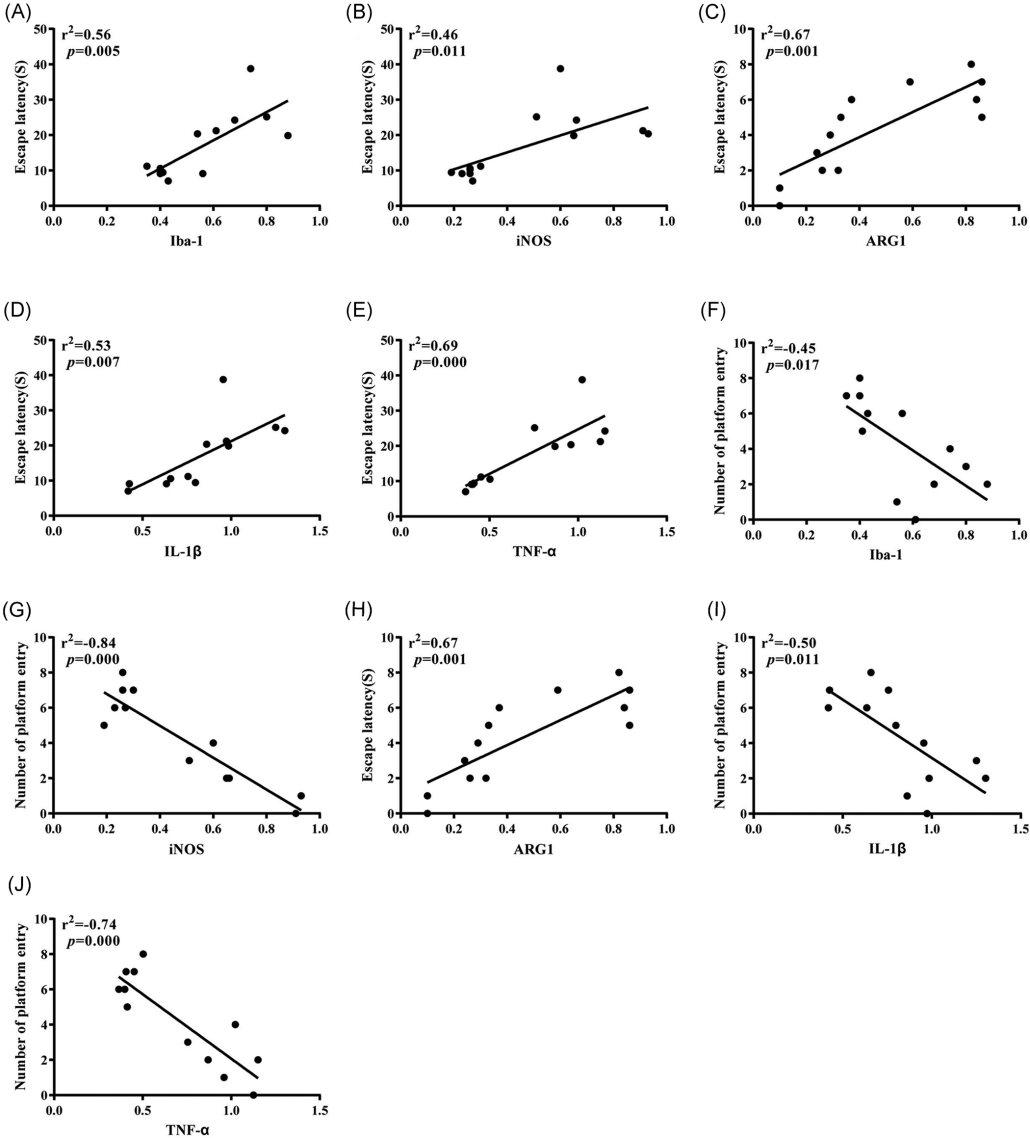
Pearson correlation analysis results. The correlation between each group of indicators was obtained in the Western blot experiment and the behavior test. (A–E) Correlation test between each index and escape incubation period. (F–J) Correlation test between each index and the times of crossing the original platform. ARG1, arginase‐1; Iba‐1, ion calcium‐binding adaptor molecule‐1; IL‐1β, interleukin‐1β; iNOS, inducible nitric oxide synthase; TNF‐α, tumor necrosis factor‐α

We further use Pearson to analyze the correlation between each group of indicators obtained in the Western blot experiment and behavioral test. It can be seen that the expression levels of Iba‐1, iNOS, IL‐1β, and TNF‐α were positively correlated with the escape latency of experimental rats and negatively correlated with the times of crossing the original platform, while the expression of ARG1 was negatively correlated with the escape latency of experimental rats and positively correlated with the times of crossing the original platform (Table 2, Figure 7A–J).

**Table 2:** Pearson correlation analysis results statistical results

	Iba‐1	iNOS	ARG1	IL‐1β	TNF‐α	Escape latency	Number of platform entry
Iba‐1	1						
iNOS		1					
ARG1			1				
IL‐1β				1			
TNF‐α					1		
Escape latency	0.59**	0.77**	−0.64**	0.80**	0.82**	1	
Number of platform entry	−0.48**	−0.68**	0.41**	−0.57**	−0.56**		1

Abbreviations: ARG1, arginase‐1; Iba‐1, ion calcium‐binding adaptor molecule‐1; IL‐1β, interleukin‐1β; iNOS, inducible nitric oxide synthase; TNF‐α, tumor necrosis factor‐α.

** At 0.0001 significant horizontal (bilateral) correlation.

## 4 DISCUSSION

POCD is a serious complication of CNS, which is often observed in elderly patients after anesthesia. Although the research on POCD is in full swing, the potential pathogenesis is still inconclusive.^8^ There is evidence that old age is an independent risk factor for POCD. Neuroinflammatory reaction, education level, and anticholinergic drugs are also some of the risk factors.^9^ It has been reported that the PFC, as a group of independent and unique subregions, is an important brain region that regulates high‐order cognitive abilities, such as spontaneous activity, attention, learning and memory, and is very sensitive to external stimuli.^10^ In this study, the positioning navigation test showed that the escape latency of the two groups of rats was obviously shortened with time and tended to be stable, suggesting that the learning and memory abilities of all rats were at the same baseline level. In addition, when we counted the swimming speed of the rats, there was no significant difference between the two groups, which indicated that some interference caused by swimming speed could be eliminated in the next behavioral test. In the following tests of space exploration and working memory, the results showed that compared with the Con group, the rats in the Sev group showed significant changes in both tests, which suggested that the elderly model rats had cognitive dysfunction after sevoflurane intervention.

Microglia are permanent immune cells in the brain, which are characterized by great heterogeneity under physiological and pathological conditions.^11^ Many studies have shown that activated microglia directly or indirectly participate in the occurrence and development of neurodegenerative diseases.^12^, ^13^, ^14^ In CNS, Iba‐1 is the most specific expression in microglia. It is also a specific marker of microglia.^15^ In this study, compared with the Con group, the expression of Iba‐1 in the PFC of the Sev group was significantly increased, and the number of microglia increased significantly, showing amoeba‐like changes. Western blot also showed that the expression of Iba‐1 protein was significantly increased, suggesting that sevoflurane can activate microglia in the cerebral cortex of aged rats. Generally, activated microglia can polarize M1 or M2 depending on their environment. M1 is a proinflammatory phenotype, and iNOS is a marker of M1, which has the ability to kill pathogens.^16^ At the same time, it destroys the integrity of neuronal tissue structure and functional stability and aggravates neuronal damage. M2 is an anti‐inflammatory phenotype, and ARG1 is a marker of M2, which can promote cell proliferation and tissue repair and play a role in protecting and restoring neurons.^17^ In this experiment, iNOS and ARG1 were measured in two groups of rats. Compared with the Con group, iNOS expression in Western blot and ELISA in the Sev group was increased, while ARG1 was decreased, with a statistical difference.

It is worth noting that microglia will continue to activate under certain stimulation and secrete inflammatory factors, such as IL‐1β and TNF‐α, which will damage neurons to a certain extent. Excessive production of inflammatory factors will lead to a vicious circle of CNS inflammation and then lead to the occurrence and development of cognitive decline.^18^, ^19^ In this study, compared with the Con group, IL‐1β and TNF‐α in the Sev group were increased in different degrees by Western blot and ELISA, which suggested that the cognitive decline of aged rats might be caused by sevoflurane triggering microglial activation, increasing M1 expression, and triggering cascade inflammation amplification effect. At last, Pearson correlation analysis of Iba‐1, iNOS, ARG1, IL‐1β, and TNF‐α with escape latency and crossing times of original platform was carried out. The results showed that Iba‐1, iNOS, IL‐1β, and TNF‐α were positively correlated with the escape latency test and negatively correlated with crossing times of the original platform, it is suggested that the increase of Iba‐1, iNOS, inflammatory factors IL‐1β, and TNF‐α will significantly increase the latency of escaping and significantly reduce the times of crossing the original platform in the water maze test. Compared with the Con group, the Sev group needs more time to find the platform, which indicates that the ability to remember the original platform position of rats decreases accordingly. In addition, we noticed that ARG1 was negatively correlated with the escape latency test and positively correlated with the times of crossing the original platform, suggesting that compared with the Con group, the escape latency time of the Sev group increased significantly and the times of crossing the original platform decreased significantly. The results further suggest that sevoflurane can induce microglia to activate and turn to M1 type; release inflammatory factors, such as IL‐1β and TNF‐α, to damage neurons; and finally affect cognitive function.

## 5 CONCLUSION

Based on these data, the effect on cell activation of microglia plays a key role in cognitive impairment in rats. Our results show that the influence of sevoflurane on the cognitive function of aging model rats may be related to M1/M2 polarization of microglia in the PFC of rats. Therefore, this study provides a new idea for the activation of microglia in the mechanism of POCD.

## CONFLICT OF INTERESTS

The authors declare that there are no conflict of interests.

## ETHICS STATEMENT

All experimental procedures were approved by the Ethical Committee of Affiliated Hospital of Zunyi Medical University (Approval No. [2019] 2‐044).

## AUTHOR CONTRIBUTIONS

Zhao‐Qiong Zhu contributed the central idea. Zhao‐Qiong Zhu and Jun‐Jie Zhou conceived and designed the experiments. Jun‐Jie Zhou analyzed most of the data. Jun‐Jie Zhou wrote the initial draft of the paper. Mei‐Qi Xu hand‐drew some of the figures and proofread the manuscript. Chao Zhang, Xin‐Xin Yang, Xiao‐Xi Zhang, Nai‐Xin Zhang, Xu Fang, Yu‐Hang Zhu, De‐Xing Liu, and Shan Xu contributed to refining the ideas, carrying out additional analyses, and finalizing this paper.

## Data Availability

The data that support the findings of this study are available upon request from the author.
